# CAR T Cells Targeting Membrane-Bound Hsp70 on Tumor Cells Mimic Hsp70-Primed NK Cells

**DOI:** 10.3389/fimmu.2022.883694

**Published:** 2022-06-01

**Authors:** Ali Bashiri Dezfouli, Mina Yazdi, Mohamed-Reda Benmebarek, Melissa Schwab, Stefanos Michaelides, Arianna Miccichè, Dirk Geerts, Stefan Stangl, Sarah Klapproth, Ernst Wagner, Sebastian Kobold, Gabriele Multhoff

**Affiliations:** ^1^ Central Institute for Translational Cancer Research Technische Universität München (TranslaTUM), Department of Radiation Oncology, Klinikum rechts der Isar, Munich, Germany; ^2^ Pharmaceutical Biotechnology, Department of Pharmacy, Ludwig-Maximilians-Universität (LMU), Munich, Germany; ^3^ Center of Integrated Protein Science Munich (CIPS-M) and Division of Clinical Pharmacology, Department of Medicine IV, University Hospital, Ludwig-Maximilians-Universität München, Member of the German Center for Lung Research Deutsches Zentrum für Lungenforschung (DZL), Munich, Germany; ^4^ Glycostem Therapeutics BV, Oss, Netherlands; ^5^ Department of Nuclear Medicine, School of Medicine, Technical University of Munich, Munich, Germany; ^6^ Institute of Experimental Hematology, Center for Translational Cancer Research (TranslaTUM), School of Medicine, Technical University of Munich, Munich, Germany; ^7^ German Center for Translational Cancer Research Deutsches Konsortium für Translationale Krebsforschung (DKTK), Partner Site Munich, Munich, Germany

**Keywords:** adoptive Immunotherapy, Hsp70, IL-2, anti-Hsp70 CAR T cells, activated NK cells

## Abstract

Strategies to boost anti-tumor immunity are urgently needed to treat therapy-resistant late-stage cancers, including colorectal cancers (CRCs). Cytokine stimulation and genetic modifications with chimeric antigen receptors (CAR) represent promising strategies to more specifically redirect anti-tumor activities of effector cells like natural killer (NK) and T cells. However, these approaches are critically dependent on tumor-specific antigens while circumventing the suppressive power of the solid tumor microenvironment and avoiding off-tumor toxicities. Previously, we have shown that the stress-inducible heat shock protein 70 (Hsp70) is frequently and specifically expressed on the cell surface of many different, highly aggressive tumors but not normal tissues. We could take advantage of tumors expressing Hsp70 on their membrane (‘mHsp70’) to attract and engage NK cells after *in vitro* stimulation with the 14-mer Hsp70 peptide TKDNNLLGRFELSG (TKD) plus low dose interleukin (IL)-2. However, a potential limitation of activated primary NK cells after adoptive transfer is their comparably short life span. T cells are typically long-lived but do not recognize mHsp70 on tumor cells, even after stimulation with TKD/IL-2. To combine the advantages of mHsp70-specificity with longevity, we constructed a CAR having specificity for mHsp70 and retrovirally transduced it into primary T cells. Co-culture of anti-Hsp70 CAR-transduced T cells with mHsp70-positive tumor cells stimulates their functional responsiveness. Herein, we demonstrated that human CRCs with a high mHsp70 expression similarly attract TKD/IL-2 stimulated NK cells and anti-Hsp70 CAR T cells, triggering the release of their lytic effector protein granzyme B (GrB) and the pro-inflammatory cytokine interferon (IFN)-γ, after 4 and 24 hours, respectively. In sum, stimulated NK cells and anti-Hsp70 CAR T cells demonstrated comparable anti-tumor effects, albeit with somewhat differing kinetics. These findings, together with the fact that mHsp70 is expressed on a large variety of different cancer entities, highlight the potential of TKD/IL-2 pre-stimulated NK, as well as anti-Hsp70 CAR T cells to provide a promising direction in the field of targeted, cell-based immunotherapies which can address significant unmet clinical needs in a wide range of cancer settings.

## 1 Introduction

Overall, there is a significant unmet clinical need for safe and more effective therapeutic strategies for patients with advanced stage solid tumors. An upsurge in its global incidence has resulted in colorectal cancer (CRC) becoming the third most common malignancy with a substantial mortality risk (1). Although autologous and allogeneic T and NK cell-based immunotherapy approaches have recently gained considerable interest for the treatment of hematological cancers (2–5), immune escape of the tumor, the immunomodulatory effects of the tumor microenvironment (TME) (6) and acquired therapy resistance limit their broader clinical application in solid tumors (7). Cytokine stimulation, together with genetic modifications of T and NK cells with chimeric antigen receptors (CAR) and other phenotypic and functional features provide promising strategies to promote and more specifically redirect the anti-tumor activities of immune effector cells (8–11). Since the lack of tumor antigen specificity and a low antigen expression density are major factors contributing to tumor immune escape, the selection of an ideal tumor-specific target is deemed essential for optimizing clinically-effective, protective and robust anti-tumor immunity (12).

The strictly tumor-specific expression of the cell membrane-bound form of the major stress-inducible 70kd heat shock protein on a large proportion of liquid and solid tumors qualifies membrane Hsp70 (‘mHsp70’) as an ideal tumor biomarker on which to base the next generation of targeted immunotherapeutic approaches (13). The mHsp70 localization on the cell surface is enabled by sphingoglycolipids such as globotriaosylceramide (Gb3) which are only present in the plasma membranes of tumor but not normal cells (14). Notably, tumor cells expressing Hsp70 on their plasma membrane actively release Hsp70 in extracellular vesicles termed exosomes (15), levels of which can be quantified in the blood of tumor patients using the Hsp70 compELISA (16). The levels of extracellular exosomal Hsp70 in the circulation of patients with cancer reflect the mHsp70 expression by their tumors and positively correlate with tumor mass, tumor grading, metastasis and potentially therapy failure (17, 18). In line with these findings, the percentage of mHsp70 expression by a patient’s tumor, as detected by flow cytometry of viable cells isolated from tumors, also is associated with advanced tumor stages in a number of cancer settings (19), and its density is higher on recurrent disease and metastases (19, 20), and after therapeutic intervention (21) compared to therapy-naïve tumors. Taken together, these unique features qualify mHsp70 as a tumor-specific target for activated and ‘alarmed’ immune cells.

Natural killer (NK) cells play a pivotal role in the innate immune response by rapidly recognizing and killing infected and tumorigenic cells followed by the downstream triggering of long-term adaptive immunity and immune memory (22). NK cells are equipped with a wide array of germline-encoded surface receptors which tune and direct their anti-tumor activity *via* a fine balance of inhibitory and activating signals in response to tumor recognition and receptor-ligand binding (23). Stress-induced signals on tumor cells can trigger NK cell activity and cytotoxicity. However, in late stage cancers, the capacity of NK cells to control tumor growth can be impaired either by antigen loss or a down regulated expression of activatory receptors induced by an immunosuppressive TME (24, 25). An *ex vivo* stimulation of NK cells under optimal culture conditions up-regulates the expression of activating receptors (e.g., C-type lectin receptors, natural cytotoxicity receptors, NCRs) and thereby promotes anti-tumor immunity (26, 27). However, although pro-inflammatory cytokines such as interleukin (IL)-2 commonly shape NK cell behavior during pathogenesis (8), high-dose IL-2 that have been administered to patients induces a broad range of clinical toxicities (28) and stimulates the immunosuppressive activity of regulatory T (Treg) cells, limiting clinical utility.

In an effort to overcome this barrier, an alternative approach is to amplify the immune activity of NK cells by incubating them with cytokine(s) alone or in combination with other stimuli *ex vivo* (29). A key prerequisite to benefit from these stimulatory signals is attributed to the pre-existing potential of NK cells to recognize tumor-specific antigens such as Hsp70 presented on the surface of tumor cells before and, even more pronounced, after therapeutic intervention (30). We have previously shown that tumor cells expressing mHsp70 can be recognized and killed using NK cells that have been activated by *ex vivo* incubation with a 14-mer Hsp70-derived peptide (TKDNNLLGRFELSG, ‘TKD’) and low-dose IL-2 (100 U/ml for 3-5 days) in a wide range of different cancer settings (31). The safety and therapeutic potential of this approach have been demonstrated in a phase I clinical trial in patients with metastatic CRC and non-small cell lung cancer (NSCLC) (32), and a phase II clinical trial in patients with inoperable stage IIIb NSCLC after radiochemotherapy (3). A potential caveat of NK cell application being their comparably short life span and low *ex viv*o expansion rate after adoptive transfer might shorten their putative activity window (33).

In contrast to NK cells, CD8^+^ cytotoxic T cells are unable to recognize and kill tumor cells expressing Hsp70 on their plasma membrane, even after stimulation with the TKD peptide and IL-2 (31, 34), but come with a proven longevity after transfer into patients for long term effects such as chronic lymphocytic leukemia (CLL) (35). The genetic engineering of T cells to express chimeric antigen receptors (CARs) targeting relevant tumor-specific antigens has delivered innovative options for novel cancer immunotherapies (36). A remarkable advantage of CAR T cells is the antigen-specific triggering of T cell-mediated cytolytic responses *via* the release of lytic proteins such as granzyme B (GrB) (34) independent of the MHC-I expression on the tumor (37), a relatively high *ex vivo* expansion rate and the high transduction efficacy of T cells. The development of CAR technology offers an opportunity to equip T cells with the capacity to recognize previously unrecognizable antigens, including mHsp70 and at the same time harness long term activity of T cells against these antigens.

A CAR construct commonly consists of an extracellular antibody-based binding domain (single-chain variable fragment, scFv) linked by a hinge region (HR) to a transmembrane domain (TMD), and or more cytoplasmic signaling domain (38). Although several FDA-approved CAR T cell products have been shown to induce a robust and durable response in patients with hematological malignancies, similar curative effects have not yet been reported for solid tumors (39). However, the identification of suitable antigens that are selectively expressed on the surface of tumors, and at an appropriate intensity, will likely expand the application of CAR T cell therapeutics in solid tumors (40). Managing on-target/off-tumor toxicity of CAR T cells will be achieved by identifying a favorable target that has no or at least limited expression on normal tissue (41).

In the present study, we hypothesized that endowing T cells with the capacity to sense and lyse mHsp70-positive tumor cells would enable persistent targeting of a broad range of advanced cancer types. We thus compared two strategies for targeting human colorectal adenocarcinoma LS174T and LoVo cell lines that differ in their mHsp70 expression pattern providing isogenic sublines as ‘high’ and ‘low’ mHsp70 expressors: *ex vivo* stimulation of human NK cells from healthy donors with the 14-mer Hsp70 peptide TKD and low-dose IL-2 and genetic engineering of T cells of the same donors with a CAR targeting mHsp70. Therefore, human peripheral blood mononuclear cells (PBMCs) isolated from the peripheral blood of healthy donors were incubated with TKD/IL-2 to stimulate NK cell reactivity against tumor cells expressing high amounts of mHsp70. In parallel, T cells from the same donors were retrovirally transduced with the anti-mHsp70-targeting CAR. We then evaluated the performance of mHsp70-targeting TKD/IL-2 stimulated NK cells and CAR T cells from the same donors against the human colorectal adenocarcinoma LS174T and LoVo cell lines with a high and low mHsp70 expression. Specificity of anti-mHsp70 CAR T cells was demonstrated by using Hsp70 knock-down cells as targets in cytotoxicity assays.

## 2 Materials and Methods

### 2.1 Cells and Cell Culture

The wild type (wt) LS174T human colorectal adenocarcinoma cell line (originally isolated from a patient with Dukes’ type B CRC) which expresses high levels of mHsp70 (Hsp70^high^) (ATCC^®^ CL-188™; ATCC, Manassas, VA, USA) and the LDHA/B lactate dehydrogenase (LDH) double knock-out (LDH^-/-^) Hsp70^low^ LS174T cell line (42, 43) were cultured in high glucose (4 g/L glucose, Sigma-Aldrich) Dulbecco’s Eagle’s Minimum Essential Medium (DMEM) containing 10% v/v heat-inactivated fetal bovine serum (FBS; Sigma-Aldrich). The wt LoVo human colorectal carcinoma cell line (originally isolated from a patient with Stage IV Dukes’ type C CRC) (Hsp70^high^) (ATCC^®^ CCL-229™; ATCC, Manassas, VA, USA) and a transient Hsp70 knock-down cell line (Hsp70^low^) (kindly provided by Glycostem Therapeutics BV, Oss, The Netherlands), was maintained in Roswell Park Memorial Institute (RPMI)-1640 (Sigma-Aldrich) containing 10% v/v FBS and puromycin (4 µg/mL, Sigma-Aldrich).

Human PBMCs isolated from the blood of different healthy donors were primarily grown in RPMI-1640 with 10% v/v FBS. The genetically engineered anti-Hsp70 CAR T cells were grown in RPMI-1640 including 2.5% v/v human serum (Sigma-Aldrich), 1% v/v non-essential amino acids (Sigma-Aldrich), 50 µM β-mercaptoethanol (Gibco), 100 IU/mL IL-2 (Chiron), and 5 ng/mL IL-15 (PeproTech). Anonymous collection and use of PBMCs from healthy donors was approved by the institutional ethical review board (44, 45).

All cell culture media were supplemented with 1% v/v antibiotics (100 IU/mL penicillin and 100 µg/mL streptomycin, Sigma-Aldrich), 2 mM L-glutamine (Sigma-Aldrich), and 1 mM sodium pyruvate (Sigma-Aldrich). The cell culture was carried out under controlled conditions at 37˚C with 95% v/v relative humidity and 5% v/v CO_2_. Cells underwent routine control for mycoplasma contamination, and their viability was tested before each experiment by trypan blue exclusion (>90%).

### 2.2 Fluorescent Protein Labelling

Labelling of recombinant Hsp70 protein, bovine serum albumin (BSA), and the cmHsp70.1 mAb which recognizes mHsp70 (46) was performed according to our standardized protocol. Briefly, freshly prepared carbonate buffer (1 M) was added to the protein solution (1 mg/mL of phosphate-buffered saline (PBS)) at a ratio of 1:10 v/v followed by 50 μL of FITC (10 mg/mL in 0.1 M carbonate buffer, Sigma). The solution was incubated in the dark with gentle overnight shaking at 4°C. After dialysis using Slide-A-Lyzer™ Dialysis Cassettes (ThermoFisher Scientific), the protein concentration and dye/protein ratio were calculated using a microplate spectrophotometer (PerkinElmer). To avoid bacterial contamination, 0.02% w/v sodium azide was included in the solution, and the final stock was stored at 4°C, in the dark. Labelling of proteins using the Alexa Fluor™ 555 Labelling and Detection Kit (ThermoFisher Scientific) was conducted.

### 2.3 Quantification of mHsp70 Expression

The expression of mHsp70 was quantified by flow cytometry using a BD FACSCalibur™ (BD Biosciences, Heidelberg, Germany). Briefly, harvested and washed cells (2x10^5^) were incubated with FITC-cmHsp70.1 monoclonal antibody (mAb; 40 µg/mL, multimmune GmbH) in flow cytometry buffer (PBS containing 10% v/v FBS) for 30 min on ice (in the dark), after which the cell pellet was washed twice and re-suspended in flow cytometry buffer containing propidium iodide viability stain (PI; 1 μg/mL, Sigma) prior to analysis. At least 2x10^4^ viable cells were acquired for each sample, from which the percentage of positive cells was determined. An isotype-matched mAb (mouse IgG1 FITC; BD Biosciences) was used as the control to define the mHsp70-positive region. The mean fluorescence intensity (MFI) was reported by subtracting the MFI of an isotype-matched control antibody from the MFI of cmHsp70.1 positively stained cells. The Quantibrite™ PE Phycoerythrin Fluorescence beads (BD, 340495) were used to quantify the number of Hsp70 molecules on the membrane per cell by setting a calibration curve relating to the MFI values. Data were analyzed using FlowJo™ software (version 10.1).

### 2.4 NK Cell Stimulation

The PBMCs were isolated from the peripheral blood of healthy donors using density gradient centrifugation. For *in vitro* activation, PBMCs (5x10^6^ cells/mL) were incubated in cell culture medium containing TKD peptide (2 µg/mL, multimmune GmbH) and low dose IL-2 (100 IU/mL) for 4 days at 37˚C, as previously described (31). The composition of unstimulated/stimulated PBMCs and the phenotype of NK cells was confirmed by flow cytometry using the following mAbs: FITC or APC-conjugated anti-CD3 (A07746; Beckman Coulter/clone SK7; BD Biosciences), APC or PE-conjugated anti-CD56 (555518; BD Biosciences/A07788; Beckman Coulter), APC-conjugated CD69 (clone L78; BD), APC or FITC-conjugated anti-CD94 (B09980; Beckman Coulter, 555888; BD Biosciences), FITC-conjugated anti-CD226 (MA5-28148; DNAM-1, Invitrogen), PE-conjugated anti-NKG2D (CD314, FAB-139P; R&D Systems), PE-conjugated anti-NKp30 (CD337, IM3709; Beckman Coulter), PE-conjugated anti-NKp44 (CD336, IM3710; Beckman Coulter), and PE-conjugated anti-NKp46 (CD335, IM3711; Beckman Coulter) monoclonal antibodies (mAbs). Samples were also stained with the relevant isotype-matched controls.

### 2.5 Anti-Hsp70 CAR Design and Transduction

The human anti-Hsp70 CAR construct (Hsp70p-CD28-CD3ζ) was based on an established CD28-CD3ζ construct using a scFv against mHsp70 derived from the sequence of the cmHsp70.1 mAb. CD3^+^ T cells were isolated from the PBMCs of healthy donors using immunomagnetic beads (CD3 MicroBeads human, Miltenyi Biotec). Transduction and expansion of T cells were performed as previously reported (47). The culture condition was as described in section 2.1.

### 2.6 Analysis of Anti-Hsp70 CAR Expression

CAR expression on primary T cells was detected by flow cytometry with a FITC-conjugated cMyc mAb (clone SH1-26E7.1.3, Miltenyi Biotec), as previously described (48). Untransduced T cells served as controls. The binding specificity of the anti-Hsp70 CAR T cells was determined by incubating the T cells with FITC-conjugated Hsp70 protein at concentrations of 1000, 500, 100, 50, 20, 10 and 2 µg/mL. FITC-conjugated BSA at the same concentrations served as a negative control.

### 2.7 Expansion and Viability of Anti-Hsp70 CAR T Cells and Stimulated NK Cells *In Vitro*

The expansion of untransduced and Hsp70 transduced CAR T cells was assessed by removing immunomagnetic beads and allowing cells to recover for 24 hours, after which 24-well plates were seeded with T cells and anti-Hsp70 CAR T cells (5x10^5^ cells per well) and their expansion rate determined by viable cell counting every three days over a period of 36 days. For NK cells, the same amount of cells was seeded in 24-well plates followed by a stimulation with either TKD/IL-2 (2 µg/mL/100 IU/mL) as used for all further phenotypical and functional analysis or with TKD/IL-2/IL-15 (2µg/mL/100 IU/mL/5 ng/mL) to mimic the culture conditions of CAR T cells. The persistence of stimulated NK cells was compared with unstimulated NK cells in a parallel culture over a period of 15 days.

### 2.8 Human Interferon (IFN)‐γ/GrB Double-Color FluoroSpot Assay

The reactivity of unstimulated and stimulated NK cells and anti-Hsp70 CAR T cells towards target cells was determined based on IFN‐γ and GrB release, as measured using a double-color FluoroSpot assay, according to the manufacturer’s instructions (CTL Europe GmBH). In brief, pre-coated wells of an ELISpot plate were washed 1x with PBS, to which was then added 100 µl of a cell suspension containing effector and target cells at various ratios (E:T 10:1, 5:1, 2.5:1, 1:1). After co-incubation for 4 and 24 hours, wells were washed twice with PBS and twice with 0.05% v/v Tween-PBS (TPBS). Subsequently, spots were developed by incubating plates with anti-human IFN‐γ/GrB detection solution (80 µl/well) for 2 hours at room temperature (RT). Finally, plates were washed 3x with TPBS, re-incubated for a further 1 h with 100 µl/well of the diluted tertiary solution followed by final 3x washing with deionized water. The plates were air-dried overnight and spots quantified using an ImmunoSpot^®^ analyzer (CTL) equipped with ELISPOT Analysis Software.

### 2.9 Confocal Microscopy

The binding of fluorescence-labelled Hsp70 to anti-Hsp70 CAR T cells was determined using confocal imaging. For this, effector cells (3x10^5^) were washed in PBS and then incubated with Alexa Fluor™ 555-conjugated Hsp70 (50 µg/mL, multimmune GmbH) and an FITC-conjugated cMyc mAb for 15 min on ice in PBS containing 5% w/v BSA (as blocking solution), after which cells were washed using ice-cold PBS. Before imaging, nuclei were incubated with DRAQ5™ far-red DNA stain (5 µM, ThermoFisher Scientific) for 5 min. Images were acquired using a confocal laser-scanning microscope (CLSM; Leica TCS SP8, Germany) equipped with Leica LAS X software.

To determine the expression of mHsp70 by LS174T cells, cells (2x10^4^ cells/well) were seeded overnight on μ-slide 8-well chamber slides (ibidi GmbH, Germany), after which the medium was refreshed by complete medium containing FITC-conjugated cmHsp70.1 mAb. Following a 20 min incubation on ice, the cells were washed twice with cold PBS and then fixed with ice-cold 4% w/v paraformaldehyde (PFA) in PBS for 15 min at RT. After an additional washing step, the filamentous actin (F-actin) and nuclei of cells were stained with rhodamine-phalloidin (1 μg/mL) and DAPI (2 μg/mL) respectively in light protected condition for 1 hour at RT before imaging.

### 2.10 Live-Cell Imaging

To track cell behavior by live-cell imaging, effector cell/target cell membranes were firstly labelled using PKH fluorescent cell linker kits according to the manufacturer’s recommendations (Sigma-Aldrich). For this, 1 mL of green (PKH67) or red (PKH26) dye solutions (5x10^-7^ M in diluent c) were rapidly added to 2x10^6^ target or effector cells suspended in 1 mL diluent c in polystyrene tubes, respectively. The reaction was stopped after 4 min by adding 2 mL FBS. PKH67-labelled target cells were obtained by centrifugation (400 g, 10 min), and re-suspended in complete medium before being transferred into a sterile tube for washing (twice) and subsequent seeding in ibidi μ-plates (24-wells) at the desired concentration per well. After 24 hours, PKH26-labelled effector cells were added to the corresponding wells (E:T 2.5:1). Cell migration and interactions were monitored over 36 hours using an EVOS™ M7000 Imaging System with 20x objective (ThermoFisher Scientific) under standard culture condition (37˚C with 100% v/v relative humidity, and 5% CO_2_). The fluorescence intensity of the target cells (green) at indicated time points was quantified by ImageJ. The corrected total cell fluorescence (CTCF) was calculated using the following formula: CTCF = integrated density - (area of selected cell x mean fluorescence of background), and normalized to the time point one hour.

### 2.11 Cell Death Analysis

Cell death was assessed by flow cytometry using Annexin V-FITC/PI apoptosis kit according to the manufacturer’s instruction (ab14085). Briefly, the target cells (2x10^5^ cells/well) were co-cultured with effector cells (E:T 1:1) for 4 hours. The cells were collected and resuspended in a binding buffer containing Annexin V-FITC and PI, followed by a 5-min incubation at RT. For flow cytometry analysis, the effector T and PBMCs cell populations were excluded by APC-conjugated anti-CD3 and APC-conjugated anti-CD45 (MHCD4505; Life technologies/clone HI30), respectively.

### 2.12 Statistical Analysis

Differences between two independent groups were evaluated by unpaired two-tailed Student’s t-tests, while difference across multiple groups by one-way ANOVA. p values were considered statistically significant as follows: ns: not significant, *p<0.05, **p<0.01, ***p<0.001, and ****p<0.0001.

## 3 Results

### 3.1 Human Colorectal Adenocarcinoma Sublines With Differential Expression of mHsp70

**Figure 1:**
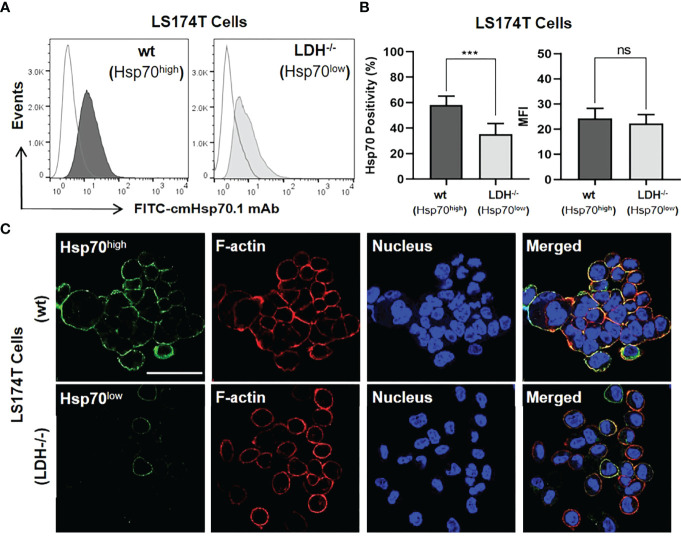
Membrane Hsp70 (mHsp70) expression on isogenic lines of LS174T cells expressing high and low densities of mHsp70 (Hsp70^high^, Hsp70^low^). **(A)** Representative flow cytometric histogram of mHsp70 expression using FITC-cmHsp70.1 monoclonal antibody (mAb). Dark (wild type, wt) and light (Lactate dehydrogenase A/B knock-out, LDH^-/-^) gray histograms. Isotype-matched (Mouse FITC-IgG1) mAb was used as negative control (white histograms). **(B)** Percentage of viable Hsp70^high^ and Hsp70^low^ LS174T wt and LDH^-/-^ cells expressing mHsp70 and MFI (mean ± SD, n≥3 independent experiments; ns, not significant; ***p ≤ 0.001). **(C)** Representative confocal microscopy images of Hsp70^high^ and Hsp70^low^ LS174T cells using FITC-cmHsp70.1 mAb (green). Nuclei were stained with DAPI (blue) and actin skeleton with rhodamine-phalloidin (red). The scale bar is 50 µm.

The outcome of a targeted immunotherapy is critically dependent on the expression profile of the tumor antigen (12, 49). As targets for our immunotherapeutic approach, we used human colorectal adenocarcinoma sublines that differ in their expression of mHsp70. As shown previously, a stable LDHA/B double knock-out of lactate dehydrogenase (LDH^-/-^) in LS174T wild type (wt) cells causes a down-regulation of the heat shock response (42). As a result, the expression of mHsp70 was stably reduced on LDH^-/-^ cells compared to LS174T wt cells, as demonstrated by flow cytometric analysis of viable tumor cells (**Figures 1A, B**) and by confocal fluorescence imaging of the isogenic tumor cell lines (**Figure 1C**). Although the LS174T cell positivity for mHsp70 was highly significant between the wt and LDH^-/-^ sublines (p ≤ 0.001), there was no remarkable difference in their MFI referring to the mHsp70 density per cell (**Figure 1B**). Hence, the mean number of mHsp70 per cell was determined using calibration beads and reported as 19648 ± 4651 for wt and 18883 ± 2475 for LDH^-/-^ cells. The co-localization of rhodamine-phalloidin (red)-labelled F-actin and mHsp70 (green) in the merged photomicrograph (yellow) demonstrates the localization of Hsp70 on the cell surface and confirms the higher expression density of Hsp70 on the membrane of LS174T wt cells compared to LDH^-/-^ tumor sublines. A transient knock-down of Hsp70 in LoVo cells generated two tumor sublines (Hsp70^high^, Hsp70^low^) that differ in their mHsp70 expression pattern (**Figure S1**). Despite a divergent expression of mHsp70, the HLA expression in both sublines was almost identical (data not shown). Both isogenic tumor cell systems were used as targets to demonstrate the mHsp70 specificity of TKD/IL-2 activated NK cells and anti-Hsp70 CAR T cells.

### 3.2 Phenotypic Characterization of PBMCs After Stimulation With TKD Hsp70 Peptide and Low Dose IL-2

**Figure 2:**
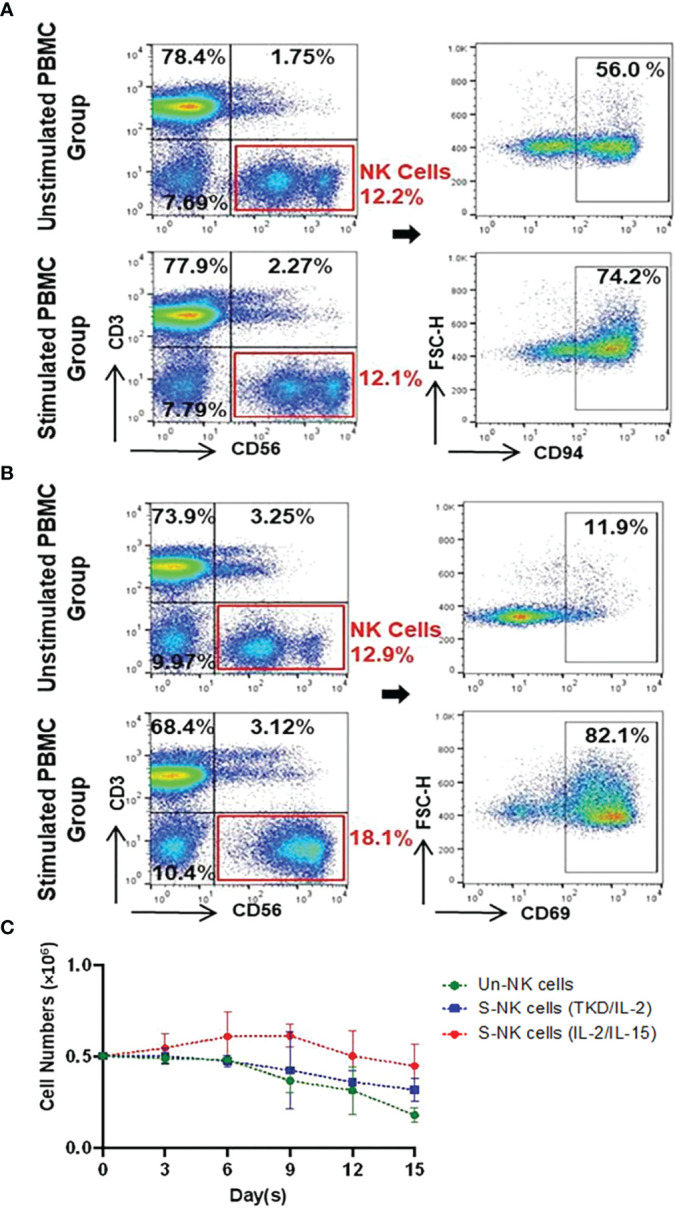
Representative gating strategy for identifying Un/S CD3^-^CD56^+^ NK cells and their expression of CD94 and CD69 as well as the persistence of NK cells. **(A)** CD94 and **(B)** CD69 gating strategies involved the identification of lymphocytes based on FSC *vs.* SSC, followed by dead cell exclusion. Within the CD3^-^CD56^+^ NK cell populations, flow cytometry plots show receptor expression relative to their counterparts incubated with the respective isotype-matched control mAb. **(C)** The persistence of unstimulated (Un) NK and stimulated (S) NK cells with either TKD/IL-2 or IL-2/IL-15 were compared in a parallel culture. After seeding (5x10^5^ cells/well), cell proliferation was determined based on viable cell counts at three-day intervals up to 15 days. Day zero of the experiment was started on the first day after removing the beads and adding to the well. Data are shown as the mean of n≥3 independent donors ± SD.

The immunophenotype of unstimulated PBMCs isolated from the peripheral blood of healthy volunteers (n=6) compare to PBMCs incubated with TKD peptide (2 μg/mL) and low dose IL-2 (100 IU/mL) for 4 days at 37°C was analyzed by flow cytometry - the results are expressed as mean percentages (± SD) and MFI of major lymphocyte subpopulations such as T cells, NKT cells and NK cells (**Table 1**). Compared to unstimulated control cells, the relative frequencies of T cells (CD3^+^/CD56^-^) and NKT cells (CD3^+^/CD56^+^) remained unchanged after stimulation with TKD/IL-2. However, a TKD/IL-2 stimulation induced a drastic up-regulation in the percentage and MFI of a large variety of activatory receptors on the CD3^-^ NK cell population, whereas none of these receptors were found to be up-regulated in the CD3^+^ T cell and NKT cell populations (data not shown). These findings are in line with previous studies showing that TKD/IL-2 stimulation selectively activates CD3^-^/CD56^+^ NK cells, but not T or NKT cell subpopulations (50). The gating strategy of the relevant unstimulated and stimulated NK cell subpopulations in PBMC preparations is illustrated in **Figures 2A, B**. The capacity of NK cells to be activated by TKD/IL-2 was found to be associated with a significant increase in the expression and density of CD69 (p ≤ 0.0001; 2.1-fold), the heterodimeric C-type lectin receptor CD94 (p ≤ 0.0001; 1.3-fold) which is involved in the binding of Hsp70 (50), CD226 (DNAM1) (p ≤ 0.01; 1.2-fold), NKG2D (p ≤ 0.001; 1.1-fold), and the NCRs (NKp30, NKp44, and NKp46; p ≤ 0.001, 1.5-fold; p ≤ 0.05, 2.2-fold; p ≤ 0.05, 1.4-fold, respectively. Moreover, the expansion capacity and persistence of NK cells isolated from PBMCs were determined *in vitro* by cell viability measurement every three days up to day 15, when cell viability of NK cells started to decline* *(**Figure 2C**). The results implied that unstimulated and stimulated NK cells followed an almost similar persistence profile between day 1 and day 15. However, a slight increase in NK cell numbers was observed after stimulation with IL-2 and IL-15 until day six, albeit afterward a downward trend occurred in all evaluated groups.

**Table 1:** Frequency of T cells, NKT cells and NK cell subsets in peripheral blood mononuclear cells (PBMCs) isolated from healthy human donors and the expression of common NK cell activating receptors on unstimulated and TKD/IL-2 stimulated NK cells.

Lymphocyte Subsets	Surface Markers	Percentage (%)			MFI		Fold Change
		Un PBMCs	S PBMCs	p Value	Un PBMCs	S PBMCs	
T cells	CD3^+^CD56^-^	75.1± 6.9	74.1 ± 6.3	ns	–	–	
NKT cells	CD3^+^CD56^+^	1.8 ± 0.84	2.1 ± 0.7	ns	–	–	
NK cells	CD3^-^CD56^+^	11.8 ± 5.2	13 ± 3.9	ns	–	–	
	CD69	13.2 ± 4.6	83.9 ± 13.3	****	356 ± 125.2	730.9 ± 192.1	2.1
	CD94	55.2 ± 6.9	82.2 ± 7.4	****	509.2 ± 114.4	652.8 ± 75.4	1.3
	CD226	80.3 ± 5.4	90.1 ± 3.9	**	55.7 ± 11	67.4 ± 19.3	1.2
	NKG2D	33.1 ± 2.03	59.1 ± 3.5	***	72.2 ± 10	77.3 ± 15.5	1.1
	NKp30	19.2 ± 5	45.1 ± 6.1	***	50.6 ± 5.7	74.5 ± 13.4	1.5
	NKp44	16.3 ± 3.1	50.3 ± 16.1	*	66.2 ± 6	144 ± 20.2	2.2
	NKp46	67.7 ± 4.9	79.1 ± 8	*	312.6 ± 67	424.4 ± 76.1	1.4

Table shows the percentage of T, NKT, and NK cells in the total PBMC population and the percentage and mean fluorescence intensity (MFI) of activating receptors (CD69, CD94, CD226, NKG2D, NKp30, NKp44, and NKp46) on Un/S NK cells ns, not significant; (*p<0.05, **p<0.01, ***p<0.001, and ****p<0.0001; mean ± SD, n≥6 independent donors). Freshly isolated PBMCs were left unstimulated (Un) or stimulated (S) with TKD (2 µg/mL) and IL-2 (100 IU/mL) for 4 days prior to the analysis of T cells (CD3^+^CD56^-^), NKT cells (CD3^+^CD56^+^) and NK cell subsets (CD3^-^CD56^+^) by flow cytometry. NK cells were further analyzed for their expression of selected surface activating receptors.

In summary, we could show that incubation of PBMCs of different donors with TKD/IL-2 significantly increased the percentage of NK cells expressing relevant activating receptors at a high density. In line with our previous findings (51), a prominent rise in the MFI of NK cells expressing CD69, CD94, NKG2D, NKG2C, NKp30, NKp44, and NKp44 was found after incubation with TKD/IL-2 but not significantly after incubation with TKD peptide alone.

### 3.3 Transduction of a Novel Anti-Hsp70 CAR Construct Into Primary T Cells

**Figure 3:**
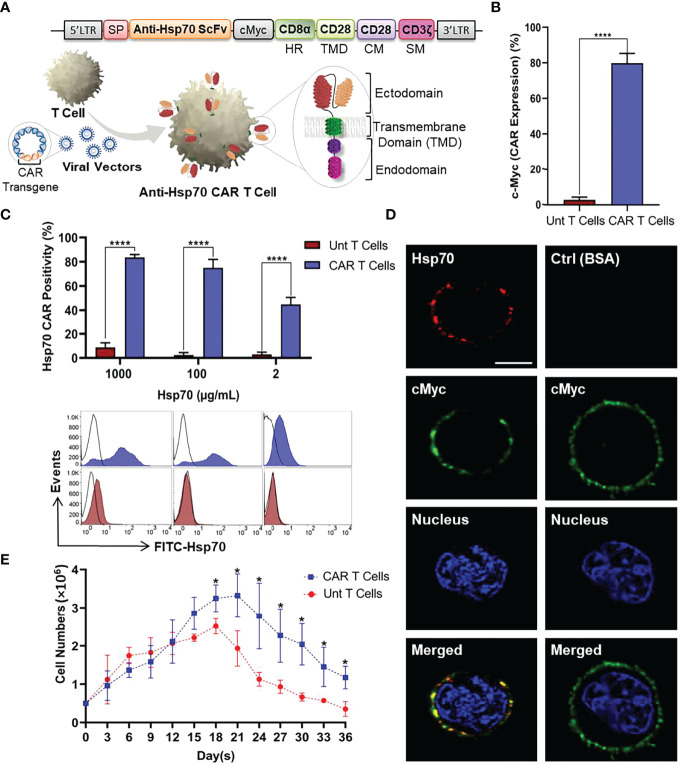
Anti-Hsp70 CAR design and engineering of primary T cells. **(A)** Schematic representation of anti-Hsp70 CAR construct and retroviral transduction into T cells isolated from PBMCs. The construct comprises of an immunoglobulin heavy-chain signal peptide (SP), single-chain fragment variant (scFv) derived from cmHsp70.1 mAb, a cMyc tag, a CD8α hinge region (HR), and a CD28 transmembrane domain (TMD) linked to an endodomain containing CD28 co-stimulatory moiety (CM) and CD3ζ signaling moiety (SM). **(B)** Anti-Hsp70 CAR transduction efficiency in T cells was analyzed by flow cytometry based on the detection of the cMyc tag by incubating untransduced (Unt) T and CAR T cells with FITC-cMyc mAb. An isotype-matched control mAb was used for setting gates. **(C)** The binding of Hsp70 protein by CAR T cells was evaluated using flow cytometry by incubating cells with FITC-Hsp70 (1000, 100, and 2 µg/mL). The bar chart shows the percentage of CAR-enriched T cells recognizing Hsp70 protein compared to Unt T cells. Histograms display the representative flow cytometry data. FITC-bovine serum albumin (BSA) was used to set the gates. The complete kinetic analysis of CAR Hsp70 protein interactions is provided in **Figure S3**. **(D)** Anti-Hsp70 CAR expression on transduced T cells was detected by confocal microscopy using Alexa Fluor™ 555-Hsp70 protein (Red) compared to cells incubated with Alexa Fluor™ 555-BSA (Red). Expression of the cMyc tag was detected using the FITC-conjugated mAb (Green). Nuclei were stained with DRAQ5™ (blue). The co-localization of Hsp70 binding and cMyc expression in the merged images appears in yellow due to overlapping green and red signals. The scale bar is 3.7 µm. **(E)** The expansion and viability of untransduced T and anti-Hsp70 CAR T cells were compared in a parallel culture. After seeding (5x105 cells/well), cell proliferation was determined based on viable cell counts at three-day intervals up to 36 days. Day zero of the experiment was started on the first day after removing the beads and adding to the well. Data are shown as the mean of n≥3 independent donors ± SD (*p ≤ 0.05, ****p ≤ 0.0001).

To investigate whether T cells can be redirected against mHsp70-positive tumor cells to utilize their demonstrated long term persistence and activity, we designed a CAR targeting mHsp70 by fusing an Hsp70-binding scFv domain derived from the cmHsp70.1 mAb (46) linked to CD28 TMD by a CD8α HR and an intracellular domain containing CD28 co-stimulatory and CD3ζ signaling moieties. The CAR was cloned into the retroviral vector pMP71. A schematic representation of the CAR cassette and retroviral transduction of T cells is illustrated in **Figure 3A**.

The manufactured anti-Hsp70 CAR T cells were subjected to various quality control assays. After transduction and removal of the stimulatory magnetic beads, the anti-Hsp70 CAR expression on T cells was evaluated by flow cytometry using a FITC-conjugated antibody directed against the c-Myc tag which is part of the extracellular domain of the anti-Hsp70 CAR construct (**Figure 3B**) or a FITC-conjugated Hsp70 protein which can be bound by the Hsp70 scFv fragment used for the anti-Hsp70 CAR. A significant proportion of the transduced (CAR) but not untransduced (Unt) T cells express the transgenic CAR on their cell surface, as detected using the FITC-conjugated anti-Myc antibody. Compared to untransduced control T cells, the anti-Hsp70 CAR T cell transduction reached an efficiency of more than 70% (mean of minimum three individual experiments ± SD) (**Figure 3B**).

We also determined the capacity of anti-Hsp70 CAR T cells to bind FITC-labelled Hsp70 protein, as a surrogate of mHsp70 expression on tumor cells. Detailed kinetic and affinity information was obtained by titrating different concentrations of FITC-labelled Hsp70 protein (1000, 500, 100, 50, 20, 10, 2 µg/mL) to anti-Hsp70 CAR T cells (**Figure S2**). Based on the flow cytometry data, a saturation in the binding of Hsp70 protein to anti-Hsp70 CAR T cells was reached at concentrations ranging between 50 and 500 µg/mL (**Figure S2**). A comparison of anti-Hsp70 CAR T cells and untransduced control cells revealed an optimal binding of Hsp70 to anti-Hsp70 CAR T cells at a concentration of 100 µg/mL; at a concentration of 1000 µg/mL an oversaturation causes non-specific binding and a concentration of 2 µg/mL an undersaturation which causes a decrease in Hsp70 binding (**Figure 3C**). Confocal microscopy was performed to confirm anti-Hsp70 CAR expression *in vitro* (**Figure 3D**; **S3**). The images clearly illustrate a co-localization of anti-cMyc (green) and labelled Hsp70 protein (red) on the cell surface of anti-Hsp70 CAR engineered effector T cells. In contrast, no red signal was detected in the control panel when anti-Hsp70 CAR T cells were incubated with identically labelled BSA (**Figure 3D**; **S3**). Furthermore, we compared the expansion rates of anti-Hsp70 CAR T cells and untransduced T cells in cell culture over a period of 36 days. On day 3 after transduction, the experiment started with 5x10^5^ viable cells from each group followed by viability measurements every three days. According to the cell expansion curve (**Figure 3E**), anti-Hsp70 CAR T cells and untransduced T cells displayed an almost identical expansion up to day 15. From day 18 onwards, the number of anti-Hsp70 CAR T cells was significantly higher than that of untransduced T cells, but the number of cells subsequently declined until day 36. Based on these findings, all further experiments were performed in the exponential growth phase of the effector cells up to day 21.

Taken together, these findings provide evidence that the anti-Hsp70 CAR was successfully transduced into T cells with desirable characteristics regarding structural and binding properties.

### 3.4 Anti-Hsp70 CAR T Cells Show Comparable Cytolytic Activity to TKD/IL-2 Activated NK Cells Against Colorectal Adenocarcinoma Cells Expressing High Levels of mHsp70

Since the release of the apoptosis-inducing serine proteases and the production of pro-inflammatory cytokines reflect the cytolytic function of lymphocytes, we assessed the release of GrB and IFN-γ in response to CRC cells expressing high and low levels of mHsp70 in a double-color FluoroSpot assay. Therefore, effector cells (TKD/IL-2 stimulated NK cells and anti-Hsp70 CAR T cells) derived from the same donors were co-cultured separately with Hsp70^high^ and Hsp70^low^ LS174T and LoVo subline target cells at different ratios (E:T) ranging from 1:1 to 10:1 for 4 and 24 hours to assess the relationship between mHsp70 density and immune target therapy. Unstimulated NK cells and untransduced T cells were used as respective controls.

**Figure 4:**
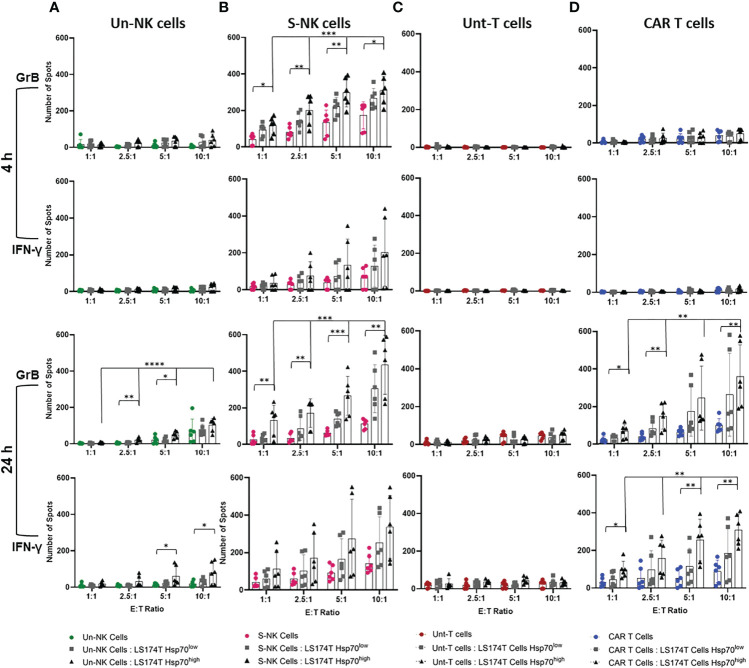
Quantification of granzyme B (GrB) and interferon (IFN)-γ release by effector cells in co-cultures with target cells. Effector cells were co-cultured with Hsp70^high^ and Hsp70^low^ LS174T target cells (E:T 1:1, 2.5:1, 5:1, 10:1). **(A)** Unstimulated (Un) NK cells, **(B)** TKD/IL-2 stimulated (S) NK cells within PBMC preparations, parallel to **(C)** untransduced (Unt) T cells, **(D)** transduced anti-Hsp70 CAR T cells. The amount of released GrB and IFN-γ was measured by double-color FluoroSpot assay after 4 or 24 hours. The number of spots at each indicated E:T ratio is represented as the mean of independent duplicates of three individual donors ± SD (*p ≤ 0.05, **p ≤ 0.01, ***p ≤ 0.001, ****p ≤ 0.0001).

As previously documented, TKD/IL-2 activated NK cells can recognize and kill tumor cells expressing mHsp70 within 4 hours of co-incubation (30). Since T cells, either unstimulated or stimulated with TKD/IL-2, are unable to recognize mHsp70 on tumor cells, an anti-Hsp70 CAR was transduced into primary T cells. The pre-stimulation of NK cells with TKD/IL-2 resulted in rapid and strong GrB release compared to the unstimulated populations at 4 and 24 hours (**Figures 4A, B**). The susceptibility of LS174T cells exhibiting a high density of mHsp70 expression to immune attack by TKD/IL-2 activated NK cells was therefore considerably greater than that of LDH^-/-^ cells with a low mHsp70 expression (**Figure 4B**). The IFN-γ FluoroSpot response was detectable after a 4-hour co-incubation period of TKD/IL-2 activated NK cell effector cells with CRC target cells, with a further increase after 24 hours of co-incubation in the individual donor.

In untransduced or anti-Hsp70 CAR transduced T cells of the same donors, no specific signs of activity-mediated secretion of GrB or IFN-γ were observed after a 4-hour co-incubation with target cells (**Figure 4C**). Even after a co-incubation period of 24 hours, untransduced T cells showed no relevant GrB or IFN-γ release (**Figure 4C**). However, anti-Hsp70 CAR T cells showed a significant ratio‐ and time‐dependent increase in GrB and IFN-γ release in response to the co-incubation with LS174T cells with a high mHsp70 expression after a 24 hour co-incubation period (**Figure 4D**). This effect was comparable to TKD/IL-2 activated NK cells after 4 and 24 hours. This effect was dose-dependent, with a higher E:T ratio causing a more pronounced release of GrB and INF-γ. ELISPOT analysis of the relative release GrB and IFN-γ supports the selective identification of Hsp70 by engineered CAR T cells which is associated with the high mHsp70 expression of LS174T wt cells. The fact that untransduced T cells did not exhibit a detectable response suggests that the anti-Hsp70 CAR T cells are ‘on-target’ (**Figures 4C, D**). Similarly, anti-Hsp70 CAR conferred an ability to transduced T cells to recognize target cells expressing mHsp70 and induce GrB release in LoVo cells (**Figure S4**).

**Figure 5:**
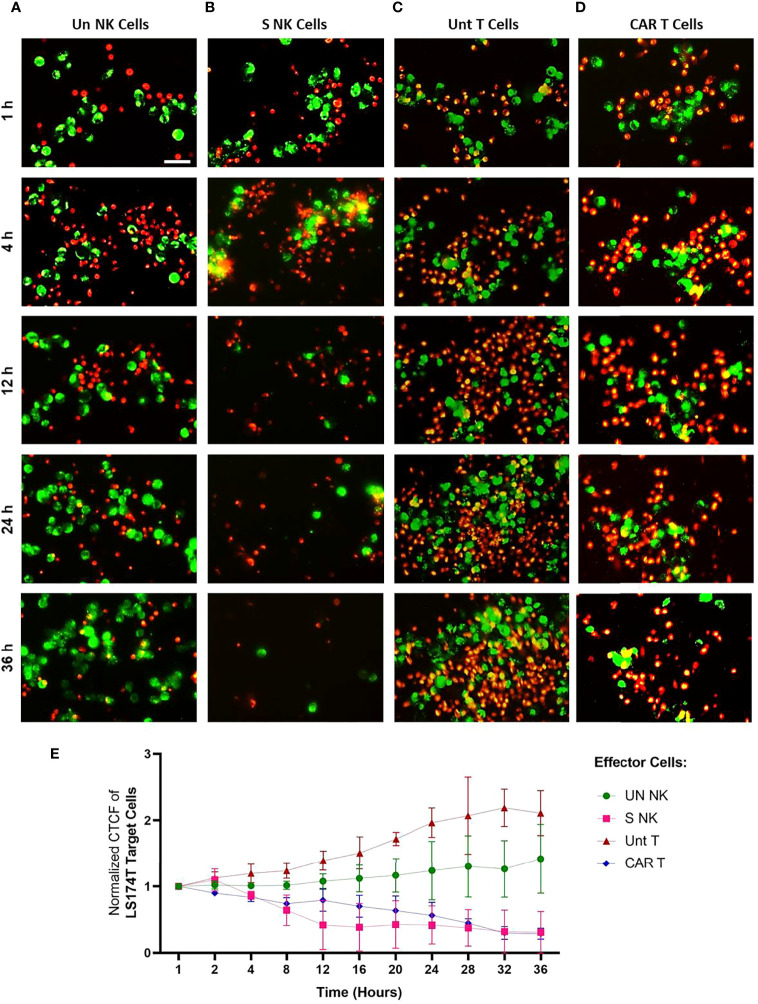
Time-lapse visualization of effector cell - target cell interactions *in vitro* using live microscopy. The interaction of effector cells against Hsp70^high^ LS174T target cells was monitored over 36 hours using time-lapse imaging. **(A)** Unstimulated (Un) NK cells, **(B)** TKD/IL-2 stimulated (S) NK cells within PBMC preparations, **(C)** Untransduced (Unt) T cells, **(D)** Transduced anti-Hsp70 CAR T cells. Effector and target cells were fluorescently stained with PKH26 (red) and PKH67 (green), respectively and co-cultured at a ratio of 2.5:1 per well of a 24-well plate. The experiment was started after adding the cells to the culture. Representative images at indicated time points (1, 4, 12, 24, and 36 hours) were provided for parallel comparison. The scale bar is 50 µm. The time-lapse images for each group are presented at various over 36 hours in **Figures S5–S8**. **(E)** The corrected total cell fluorescence (CTCF) at the following time points (1, 2, 4, 8, 12, 16, 20, 24, 28, 32, and 36 hours) was calculated as described in methods (section 2.10). The data were normalized to the time point 1 hour and presented as the mean of three independent microscopic fields ± SD.

Live imaging was performed to visualize the cytotoxic activity of NK and anti-Hsp70 CAR T cells to tumor cells expressing high levels of mHsp70 and their cellular fates over time. The mHsp70^high^ expressing LS174T cell line (green) was co-cultured with TKD/IL-2 stimulated NK cells or anti-Hsp70 CAR T cells (both red) at an E:T ratio of 1:2.5, with unstimulated NK cells and untransduced primary T cells serving as controls. The effector activity against tumor target cells was monitored every 15 min for a time interval of 36 hours. Representative images of the effector-to-target cell interactions are shown after a 1, 4, 12, 24, and 36 hour co-incubation time (**Figure 5**). Additional images of all co-incubation sets after shorter time intervals are provided in **Figures S5-S8**. A co-incubation of unstimulated NK cells (**Figure 5A**) and untransduced T cells (**Figure 5C**) did not result in any relevant tumor cell killing over a period of 36 hours; the actual number of tumor cells (green) increased over this time period (**Figures 5A, C**). In contrast, a clear reduction in tumor cells was observed after 4 to 12 hours when they were co-incubated with TKD/IL-2 activated NK cells (**Figure 5B**), after 24 hours when they were co-incubated with anti-Hsp70 CAR T cells (**Figure 5D**), and after 36 hours nearly no viable tumor cell was visible. The specific anti-tumor activity of activated NK cells and anti-Hsp70 CAR T cells was evident by microscopic examination. Of particular interest is that TKD/IL-2 pre-stimulation yielded a robust degranulation of GrB, leading to a superior and fast functionality of NK cells already after 4 hours, whereas anti-Hsp70 CAR T cells showed their highest lytic potential against mHsp70-positive LS174T cells after 24 hours. As the first line of defense, NK cells that - after stimulation- are equipped with a wide range of germline-encoded activatory surface receptors have the capacity to recognize and kill their targets much faster than T cells. The cytotoxicity assessment by Annexin/PI staining method confirms the fast and efficient response of TKD/IL-2 activated NK cells against mHsp70-positive tumor cells (**Figure S9**). At later time points, the clustering of stimulated NK cells and anti-Hsp70 CAR T cells is more pronounced, highlighting the time dependence of the specificity for killing target cells expressing mHsp70. Of note, the mHsp70 positivity on tumor cells was reduced 1.44 ± 0.1 fold after a 36 hour co-incubation with anti-Hsp70 CAR T cells and 1.5 ± 0.3 fold after a 24 hour co-incubation with TKD/IL-2 activated NK cells. These data demonstrate the mHsp70 specificity of tumor cell killing by anti-Hsp70 CAR T cells and activated NK cells. In contrast, unstimulated NK cells and primary T cells did not show microscopic signs of effector-to-target cell interactions nor any lytic activity over time, and allowed even the proliferation of cancer cells over the time period of 36 hours (**Figure 5E**).

## 4 Discussion

The role of cellular immunotherapies in tumor surveillance and control has become more widely appreciated in recent years. Since the solid tumor landscape presents diverse and complex resistance mechanisms, rendering many approaches less effective, intensive investigations are focused on approaches to promote, genetically or otherwise, the functionally and the anti-tumor effects of adoptive cells (52). For this particular aim, we used two different strategies: firstly, we addressed activating receptors responsible for NK cell stimulation triggered by an interaction of NK cells with their corresponding tumor antigen TKD in the presence of pro-inflammatory cytokines (IL-2). Secondly, primary T cells were engineered with viral vectors encoding for an anti-Hsp70-specific CAR with the aim to endow these, potentially long living cells, with Hsp70 recognition capacity and trigger active tumor clearance. Since the antigen-receptor interaction is the critical aspect of the strategies applied in this study, selecting the best antigen with sufficient affinity, specificity, density and valence is of utmost importance. The stress-inducible mHsp70 on tumor cells exquisitely meets the criteria as an ideal target for our utilized strategy. Herein, we have concurrently expanded the potential portfolio of therapeutics targeting tumors mHsp70 expression from the established recognition by TKD/IL-2 activated NK cells to include CAR T cells that have been re-directed to target Hsp70 by genetic cell bioengineering. As illustrated in this study, despite the different kinetics, stimulated NK cells and anti-Hsp70 CAR T cells both showed promising Hsp70-targeting capacities

To reach an optimal immune activity of NK cells against solid tumor-related adoptive resistance, several strategies have been developed for regulating the involved receptors, for example, by a targeted blockade of inhibitory receptors or ligation of the activating receptors (53). With a similar purpose, extensive studies have employed pro-inflammatory cytokines such as IL-2 either in *ex vivo* expansion and activation approach or *via* systemic injection (54, 55). Cytokines, either alone or as cocktails favor the prolonged and elevated cytotoxic activity of NK cells and contributes to improved cancer patient survival rates (56, 57). Despite clinical success using high and frequent doses of IL-2, cytokine-associated toxicities such as vascular leak syndrome, hypotension, and liver toxicities need to be considered, particularly when administered systemically (58). Moreover, high doses of IL-2 can adversely affect the efficacy of adoptive T cell therapies in a dose-dependent manner by driving the inhibitory effects of CD4^+^/CD25^+^/FoxP3^+^ regulatory T (Treg) cells in patients (59, 60). However, on a positive side, the impaired functionality of NK cells isolated from the peripheral blood of patients with CRC can be recovered by either IL-2 or IL-15 and when combined with Cetuximab enables antibody-dependent cellular cytotoxicity (ADCC) (61).

It is essential to identify optimal protocols for NK cell stimulation, especially when developing therapies for malignancies that are inherently resistant to autologous cell-based immunotherapies (62–64). In line with our previous results (32), we confirmed the immunostimulatory potential of *ex vivo* stimulation of NK cells using the TKD Hsp70 peptide in combination with low dose IL-2. TKD is a 14-mer peptide sequence derived from the C-terminal binding domain of Hsp70 which is exposed on the cell surface of tumor cells. In combination with IL-2, TKD has identical activation properties to the full-length 70kD-Hsp70 protein (65). The advantage of our method which involves the activation of PBMCs is that there is no requirement for NK cell purification by, for example, the magnetic bead separation methods which can cause effector cell loss. Additionally, this procedure benefits from reproducibility and replicability. Since cytokine-mediated toxicities mostly come from the high-dose IL-2 application *in vivo* (28), a protocol involving *ex vivo* use of low dose IL-2 avoids adverse effects. The synergistic effect of the TKD/IL-2 on NK cell stimulation, compared to IL-2 alone, has been documented by an increased production of GrB and IFN-γ and interaction with the tumor target cells.

The potency of TKD/IL-2 activated NK cells to recognize Hsp70 (30) depends on the nature of the target cells and their expression of mHsp70. Cells expressing mHsp70 generate signals that induce the migration of activated NK cells and enable infiltration into solid tumors (66). Consistent with this finding, we demonstrate that TKD/IL-2 activated NK cells preferentially migrated towards mHsp70^high^ (as opposed to Hsp70^low^ data not shown) LS174T CRC cells. As expected, this resulted in NK cell binding to target cells and the triggering of effector cell cytotoxicity-related events, such as the release of effector molecules, including GrB, and induces apoptosis in cancer cells expressing mHsp70 (67). Cytotoxicity has been shown to occur *via* a perforin-independent apoptosis which involves mHsp70 mediated uptake of GrB (34). In view of the relationship between functionality and an increased expression of activating receptors, we found a clear and consistent influence of TKD/IL-2 stimulation on the expression of NKG2D, a member of the NKG2 family, members of the NCR family (NKp30, NKp44, and NKp46), DNAX accessory molecule-1 (DNAM-1; CD226), CD69, and CD94 by NK cells from different healthy human donors. Ligation of the NKG2D receptor is a dominant trigger in the activation status of the NK cells which can circumvent signals transmitted through the ligation of inhibitory receptors (68). NKG2D works together with NCRs in a complementary or synergistic fashion reflected by an increase in the cytolytic ability (69). DNAM-1 also belongs to the major killer receptors that generate NK cell-activating signals (70). The expression of CD69 and CD94 were assessed due to their role as a rapidly expressed activation marker and their interaction with Hsp70, respectively. In connection with the proven interplay between CD94 and Hsp70, we have previously demonstrated the relationship between the upregulation of CD94 on NK cells after TKD/IL-2 stimulation and their cytotoxic function (71, 72). To date, NK cells from patients with CRC have been reported to express reduced levels of common activating receptors and a correspondingly impaired function (70, 73). Hence, it is important to overcome these functional deficiencies and activate the appropriate signaling network in adoptively transferred NK cells in cancers such as CRC. Our results focus on the remodeling of the NK cell phenotype and function in the area of tumor antigen-dependent immunotherapies. Stimulation of NK cells with TKD/IL-2, significantly increased the proportion of NK cells expressing relevant receptors, and the density of expression, albeit to varying degrees. Although the current study has employed PBMCs obtained from healthy donors, similar responses have been observed when using cells obtained from patients with cancer (3). Taken together, TKD/IL-2 pre-stimulation of NK cells and their subsequent interaction with tumor cells expressing mHsp70, influences the expression of functional receptors and triggers an effective anti-tumor cytotoxic response, the potency of which is dependent on the expression of mHsp70.

The genetic engineering of immune effector cells such as T and NK cells to express defined CARs is a component of current frontier technologies driving innovative cancer immunotherapies (36, 49). The CAR structure brings advantages in terms of efficiency and safety which stem from the specified targeting of tumor antigens in an MHC-I-independent manner and the enhanced expansion and persistence of effector cells resulting in persistent responses (37, 74). CAR T cells have provided unprecedented success in hematological malignancies, with FDA approval for relapsed and refractory (R/R) B-cell acute lymphoblastic leukemia (B-ALL) (75), mantle cell lymphoma (76), diffuse large B-cell lymphoma (77), and R/R multiple myeloma (MM) (78). However, CAR T cells have not yet delivered the same levels of efficacy in the setting of solid tumors. The immunosuppressive status of the tumor microenvironments (TMEs) overcome the capacity of native and CAR-enriched T cells to control tumors (79). To overcome this, a wide range of CARs against multiple antigens that are expressed by CRC (GUCY2C (80), EpCAM (81), NKG2D (82), Her2 (83), MUC (84), etc.) are being evaluated using *in vitro* and pre-clinical animal models. Although tumor trafficking and infiltration occur, several antigen-related barriers (e.g., antigen heterogeneity or loss, or unavailability on the cell surface) limit the powerful ability of CAR T cells to selectively target desired antigens (85). Moreover, on-target and off-tumor toxicity resulting from nonspecific targeting of normal tissue antigens is among the major issues that currently hinder the safety of CAR T cell therapy (86, 87). For example, a patient with metastatic CRC experienced pulmonary complications and subsequent death after an infusion of HER2 CAR T cells which was likely attributable to the expression of HER2 in the lung epithelium (87). Therefore, one possible way to overcome these challenges is selecting a target antigen which is specifically expressed on tumor cells, exhibiting a sufficient binding affinity and an appropriate distribution on the cell surface (88).

The membrane form of Hsp70 is a promising antigen for immune targeting due to its selective and specific expression on tumors such as CRC, while being undetectable in corresponding normal cells and tissue (13, 89, 90). In addition to this notable advantage, this stress-inducible protein can be recognized by anti-Hsp70 CAR T cells. Antibody-mediated targeting of Hsp70 on tumor cells has been successfully applied for the treatment of CRC and other malignancies (91). Herein, we genetically engineered primary T cells to recognize mHsp70 on cancer cells by retrovirally inserting the anti-Hsp70 CAR cassette, and demonstrated that the CAR is expressed at sufficient level and triggers lytic signaling. In this construct, the anti-Hsp70 scFv is derived from cmHsp70.1 mAb. We have previously reported this antibody to efficiently bind mHsp70 and inhibit tumor growth in mice bearing CT26 colorectal adenocarcinoma (46). The advantage of the approach lies in enhanced infiltration into solid tumors and selectively elimination of tumor cells expressing mHsp70. The design of the CAR structure plays a fundamental role in the success (38). Our second-generation CAR is composed of CD8α-derived HR and TMD linking the Hsp70 scFv ectodomain to the CD3ζ signaling endodomain. Besides the structural aspect, HR and TMD types are potentially important for the level of CAR expression and CAR-mediated function, despite differing target antigens (92–94). The CD28 co-stimulatory domain not only amplifies the signaling induced by the CD3ζ domain (95, 96), but also positively influences the clinical persistence of CAR T cells (97). Based on the currently available data, our ability to confirm sufficient level of expression and the binding specificity of the anti-Hsp70 CAR on T cells provides evidence for their therapeutic potential. The improved *in vitro* persistence of anti-Hsp70 CAR T cells compared to normal T cells sheds light on possible long-term persistence in clinical scenarios. The anti-tumor activity of CAR T cells was demonstrated by an increased GrB degranulation and IFN‐γ secretion which translated into enhanced cytotoxicity of CRC cells expressing mHsp70. Based on the data from the CRC cell lines the cytotoxicity of the anti-Hsp70 CAR T cells is related to the amount of mHsp70 expressing cells.

## 5 Conclusion

Based on this systematic *in vitro* study, we have highlighted the importance of mHsp70 as a target on highly aggressive tumors and the therapeutic potential of anti-Hsp70 CAR T cells. The exclusive expression of Hsp70 on tumor but not normal cells, and the broad expression of mHsp70 on many different tumor entities supports the broad applicability, specificity and safety of the approaches described herein, given the reduced likelihood of off-target effects, especially in the context of anti-Hsp70 CAR T cells. Considering the safety and efficacy aspects, TKD/IL-2 activated autologous NK cells have already been shown to have a favorable safety and efficacy profile in a phase II clinical trial in patients with advanced NSCLC (3).

In summary, we have been able to transduce PBMC-derived T cells with a novel anti-Hsp70 CAR and thereby enable them to recognize CRC cells expressing mHsp70 in a way that resembles the specificity and cytolytic activity of TKD/IL-2 activated NK cells. Exposure of TKD/IL-2 stimulated NK cells and anti-Hsp70 CAR T cells to CRC cells expressing mHsp70 triggers GrB-mediated cytotoxicity and IFN-γ release which causes tumor control *in vitro*. Further studies are required to assess this novel treatment in relevant *in vivo* models.

## Data Availability Statement

The raw data supporting the conclusions of this article will be made available by the authors, without undue reservation.

## Ethics Statement

The studies involving human participants were reviewed and approved by institutional ethical review board. Written informed consent for participation was not required for this study in accordance with the national legislation and the institutional requirements.

## Author Contributions

ABD, MY performed and analyzed data, wrote the original draft of the manuscript (Ms) and provided the Figures. M-RB, SM produced CAR T cells; MS, SS, SKL performed and analyzed data. AM, DG produced knock-down cells; EW, SKL proof-read the Ms. GM conceptualized and designed the study, and proof-read the Ms. All authors have read and agreed to the published version of the manuscript.

## Funding

ABD is supported by an Alexander von Humboldt Research Fellowship for his postdoctoral at Technische Universität München; MY is supported by a DAAD fellowship for her Ph.D. study at Ludwig-Maximilians-Universität Munich; this study was supported by the DFG (Grant KU3500/2-1, to MS; STA1520/1-1, to SS; SFB824/3-3, to GM); Marie-Sklodowska-Curie Program Training Network for Optimizing Adoptive T Cell Therapy of Cancer funded by the H2020 Program of the European Union (Grant 955575, to SK); by the Hector Foundation (to SK); by the International Doctoral Program i-Target: Immunotargeting of Cancer funded by the Elite Network of Bavaria (to SK); by Melanoma Research Alliance Grants 409510 (to SK); by the *Else Kröner-Fresenius-Stiftung* (to SK); by the German Cancer Aid (to SK); by the Ernst-Jung-Stiftung (to SK); by the LMU Munich’s Institutional Strategy LMUexcellent within the framework of the German Excellence Initiative (to SK); by the *Bundesministerium für Bildung und Forschung* (to SK and GM); by the European Research Council Grant 756017, ARMOR-T (to SK); by the German Research Foundation (DFG) (to SK); by the SFB-TRR 338/1 2021–452881907 (to SK); by the Fritz-Bender Foundation (to SK), by the BMWi (ZF4320104AJ8, to GM) and by the José-Carreras Foundation (to SK).

## Conflict of Interest

GM. is the founder and Chief Scientific Officer of multimmune GmbH.

SK has received honoraria from TCR2 Inc, Novartis, BMS and GSK.

SK is an inventor of several patents in the field of immuno-oncology.

SK received license fees from TCR2 Inc and Carina Biotech.

SK received research support from TCR2 Inc. and Arcus Bio-science for work unrelated to the manuscript.

AM and DG are employed by Glycostem Therapeutics BV.

The remaining authors declare that the research was conducted in the absence of any commercial or financial relationships that could be construed as a potential conflict of interest.

## Publisher’s Note

All claims expressed in this article are solely those of the authors and do not necessarily represent those of their affiliated organizations, or those of the publisher, the editors and the reviewers. Any product that may be evaluated in this article, or claim that may be made by its manufacturer, is not guaranteed or endorsed by the publisher.
