# Supplementary material for: CAR T Cells Targeting Membrane-Bound Hsp70 on Tumor Cells Mimic Hsp70-Primed NK Cells
Source: Front Immunol. 2022 Jun 1;13:883694. doi: 10.3389/fimmu.2022.883694 (PMC9198541; doi:10.3389/fimmu.2022.883694)
Supplement: Supplementary file 1 [file DataSheet_1.pdf]

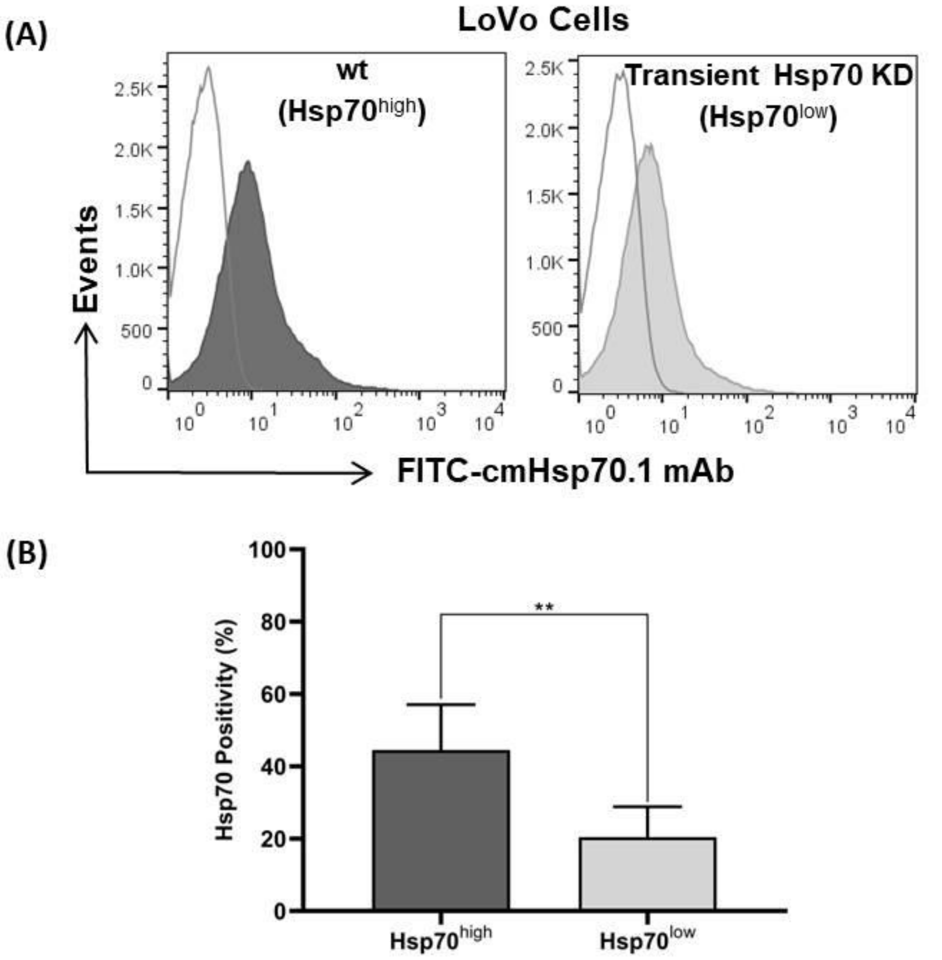

Figure S1. Membrane Hsp70 (mHsp70) expression on isogenic lines of LoVo cells expressing high and low densities of mHsp70 (Hsp70^{high}, Hsp70^{low}). (A) Representative flow cytometric histogram of mHsp70 expression on viable cells using the FITC-cmHsp70.1 monoclonal antibody (mAb) (Dark (wild type, wt) and light (transient Hsp70 knock-down (KD), Hsp70$^{-/-}$) gray histograms). Isotype-matched (Mouse FITC-IgG1) mAb was used as negative control (white histograms). **(B)** Percentage of viable Hsp70^{high} and Hsp70^{low} LoVo wt and Hsp70$^{-/-}$ cells expressing mHsp70 (mean ± SD, n≥3 independent experiments; **p≤0.01).

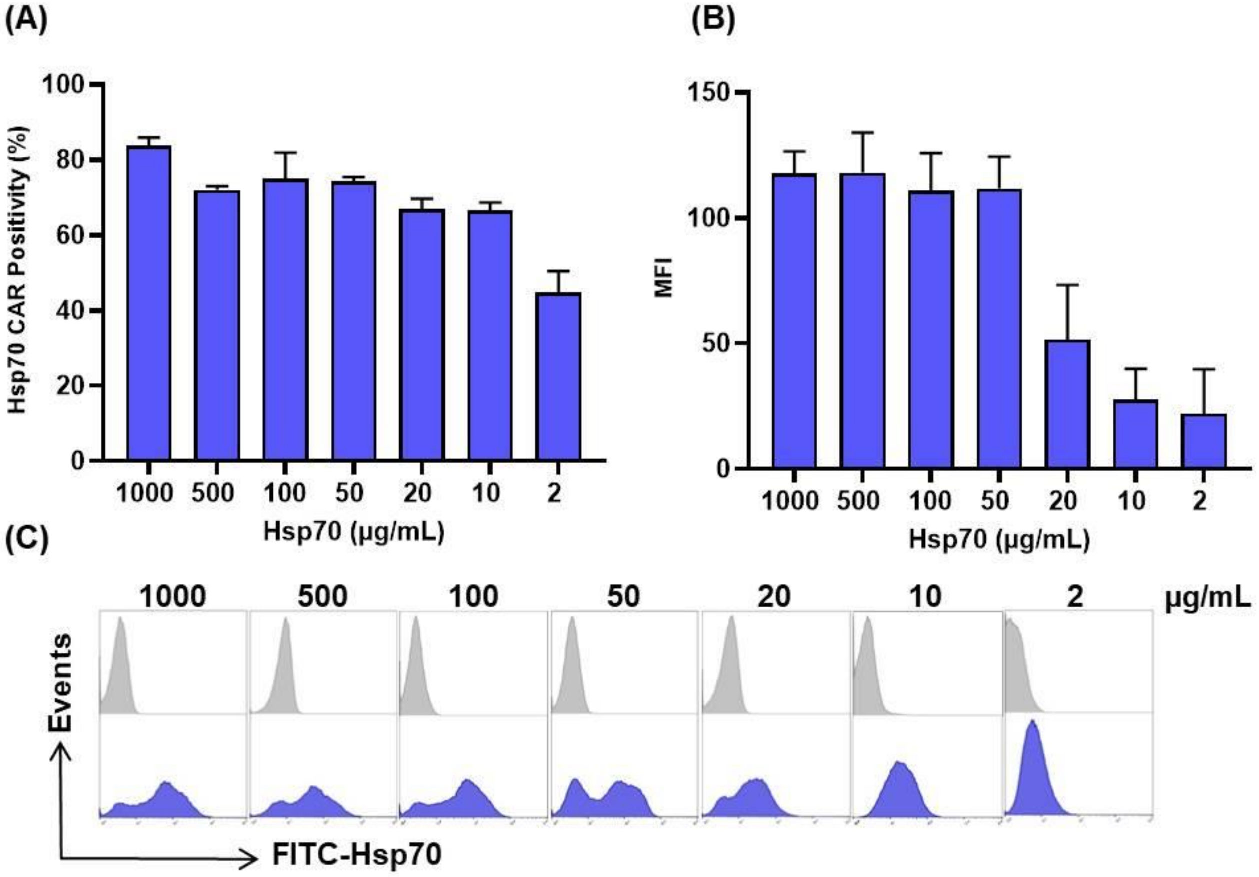

Figure S2. Kinetics of Hsp70 protein - CAR construct interactions. The binding of FITC-Hsp70 (1000, 500, 100, 50, 20, 10, and 2 µg/mL) to anti-Hsp70 CAR T cells was evaluated by flow cytometry. The bar chart shows the percentage **(A)** and MFI **(B)** of anti-Hsp70 CAR-enriched T cells binding Hsp70 protein cells (mean ± SD, n≥3 independent donors). **(C)** Histograms display the representative flow cytometry data. FITC-BSA was used to set the gates.

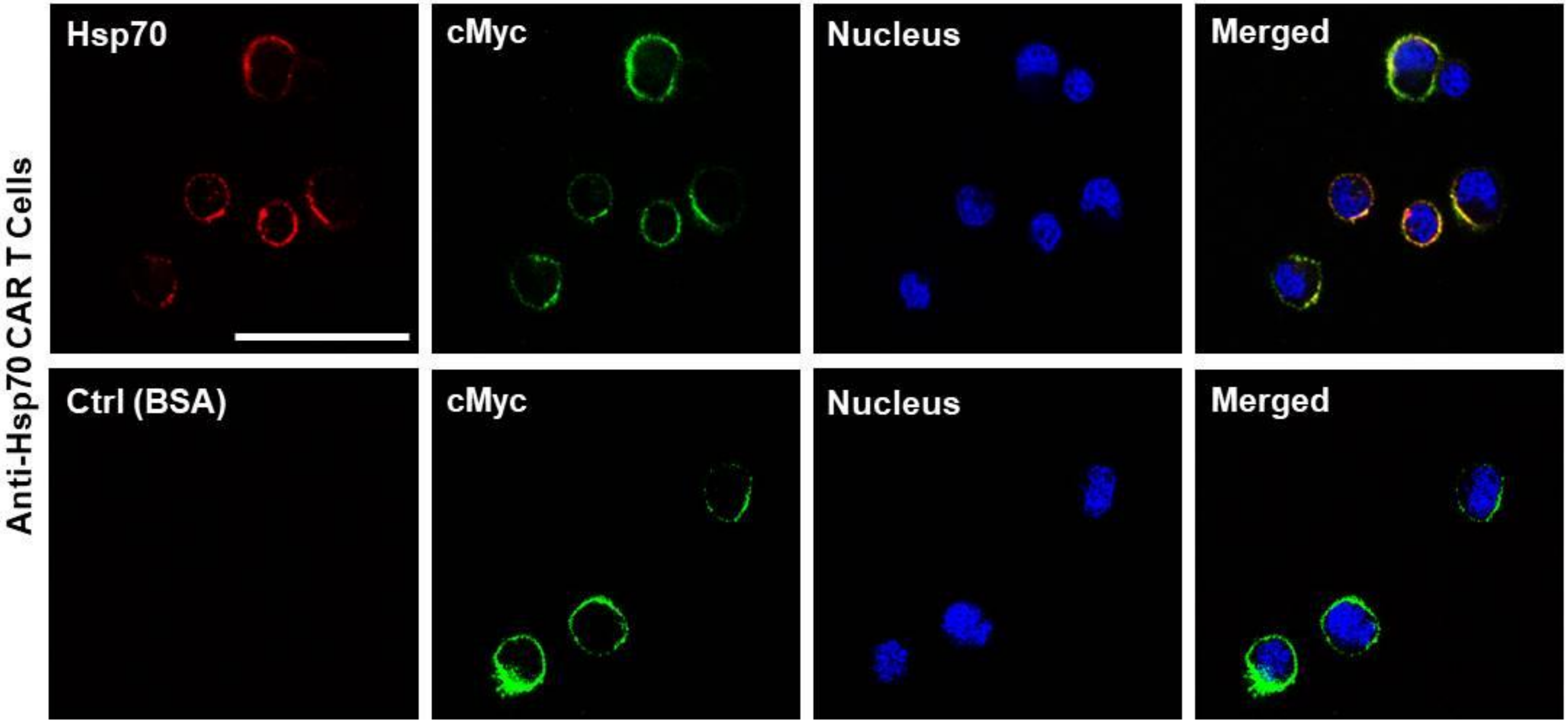

Figure S3. Surface expression of anti-Hsp70 CAR on virally transduced T cells. The anti-Hsp70 CAR construct was virally transduced into primary T cells isolated from PBMCs. The anti-Hsp70 CAR expression on transduced T cells was detected by confocal microscopy using Alexa Fluor 555™-Hsp70 protein (red), using Alexa Fluor 555™-BSA as controls. The presence of the cMyc tag was detected using a FITC-conjugated mAb (green). Nuclei were stained with DRAQ5™ (blue). The co-localization of Hsp70 binding and cMyc expression appears in yellow due to overlapping green and red signals. The scale bar is 36.8 µm.

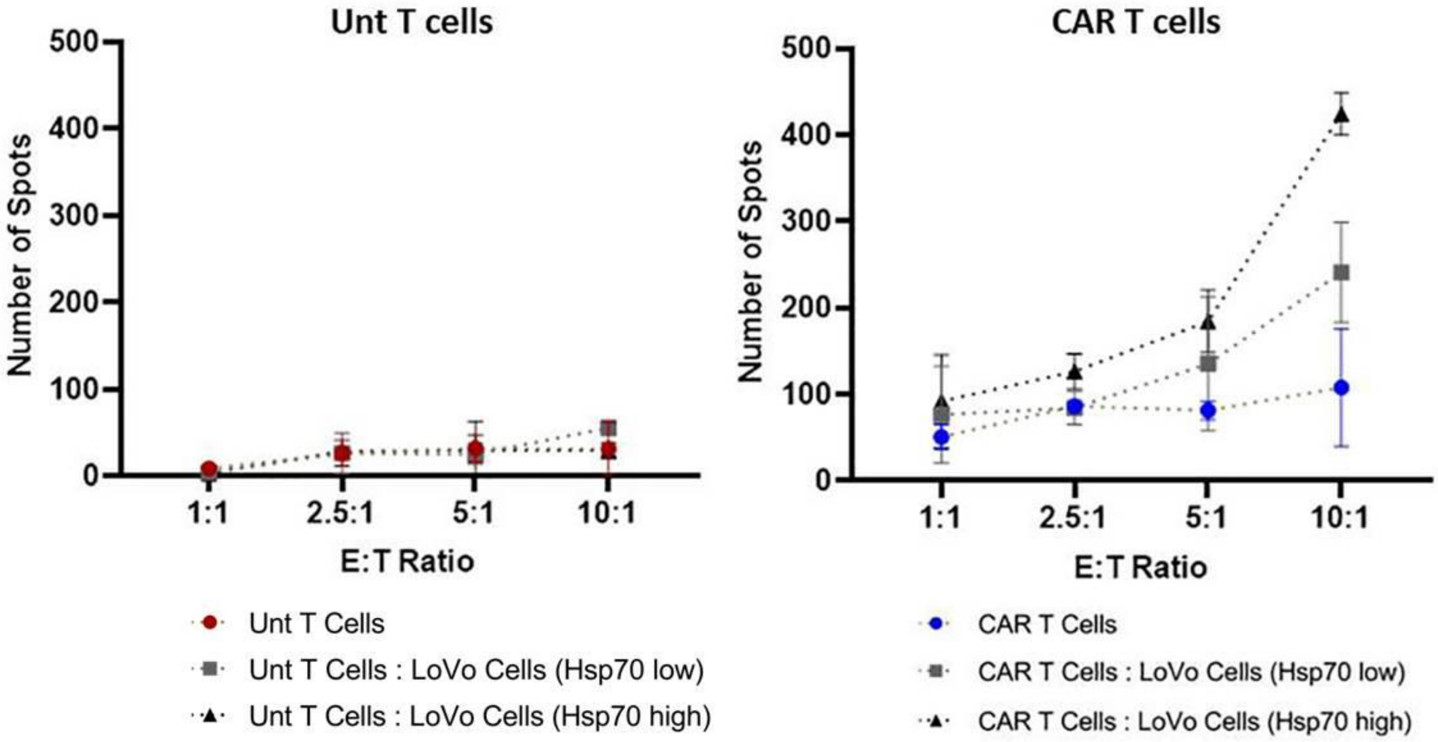

Figure S4. Quantification of granzyme B (GrB) and interferon (IFN)-γ release by effector cells in co-cultures with target cells. Effector cells were co-cultured with $Hsp70^{high}$ and $Hsp70^{low}$ LoVo target cells (E:T 1:1, 2.5:1, 5:1, 10:1). **(A)** Untransduced **(**Unt) T cells, **(B)** Transduced anti-Hsp70 CAR T cells. The amount of released GrB and IFN-γ was measured by double-color FluoroSpot assay after 24 hours. The number of spots at each indicated E:T ratio is represented as the mean of duplicates of three independent donors ± SD.

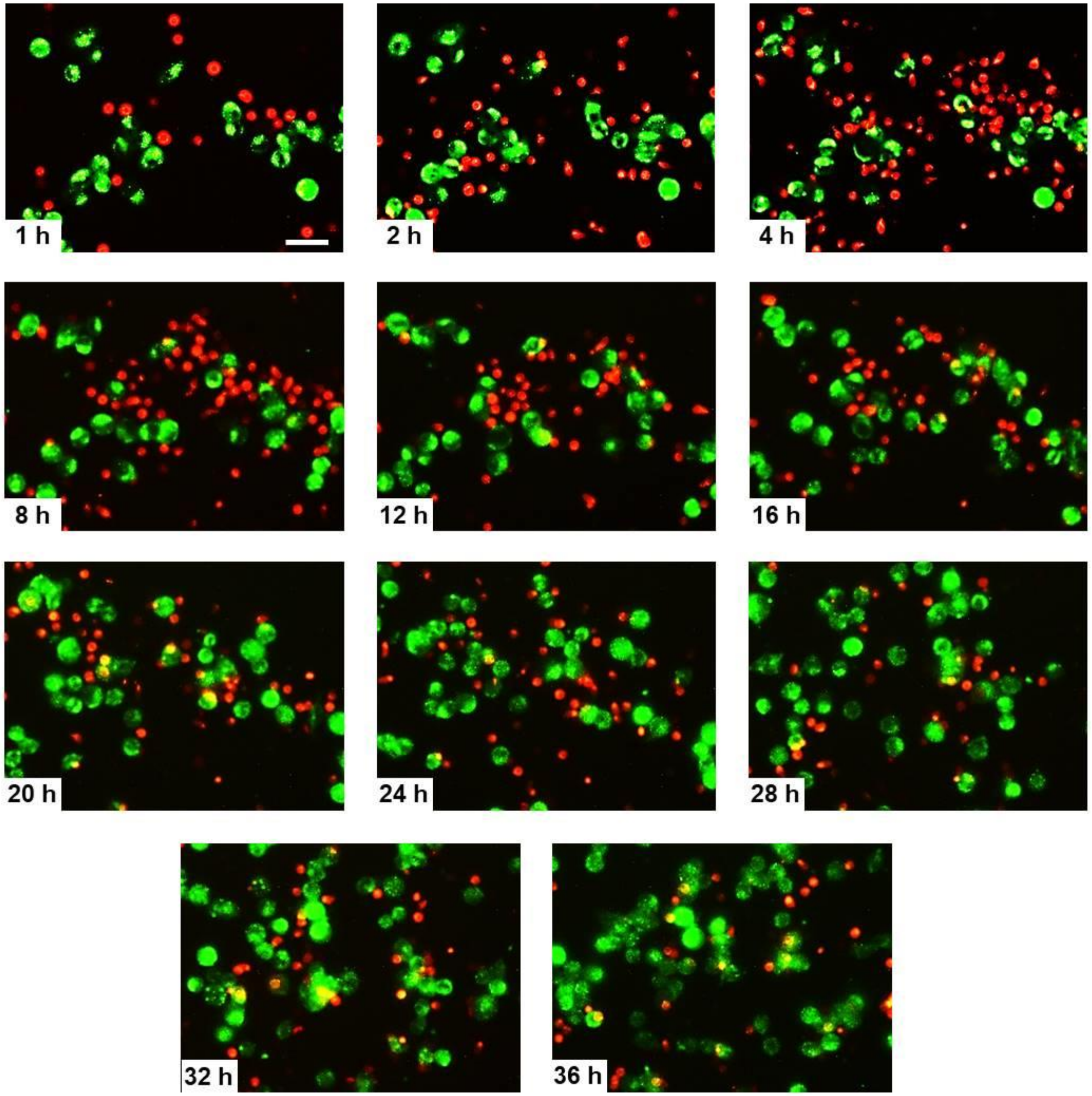

Figure S5. Time-lapse visualization of effector cell - target cell interactions *in vitro* using live microscopy. The interaction of unstimulated (Un) NK cells within a PBMC preparation (red) with Hsp70^{high} LS174T target cells (green) (E:T 2.5:1) was monitored by a time-lapse imaging system at various intervals over 36 hours. The experiment was started after adding the cells to the culture. The scale bar is 50 µm.

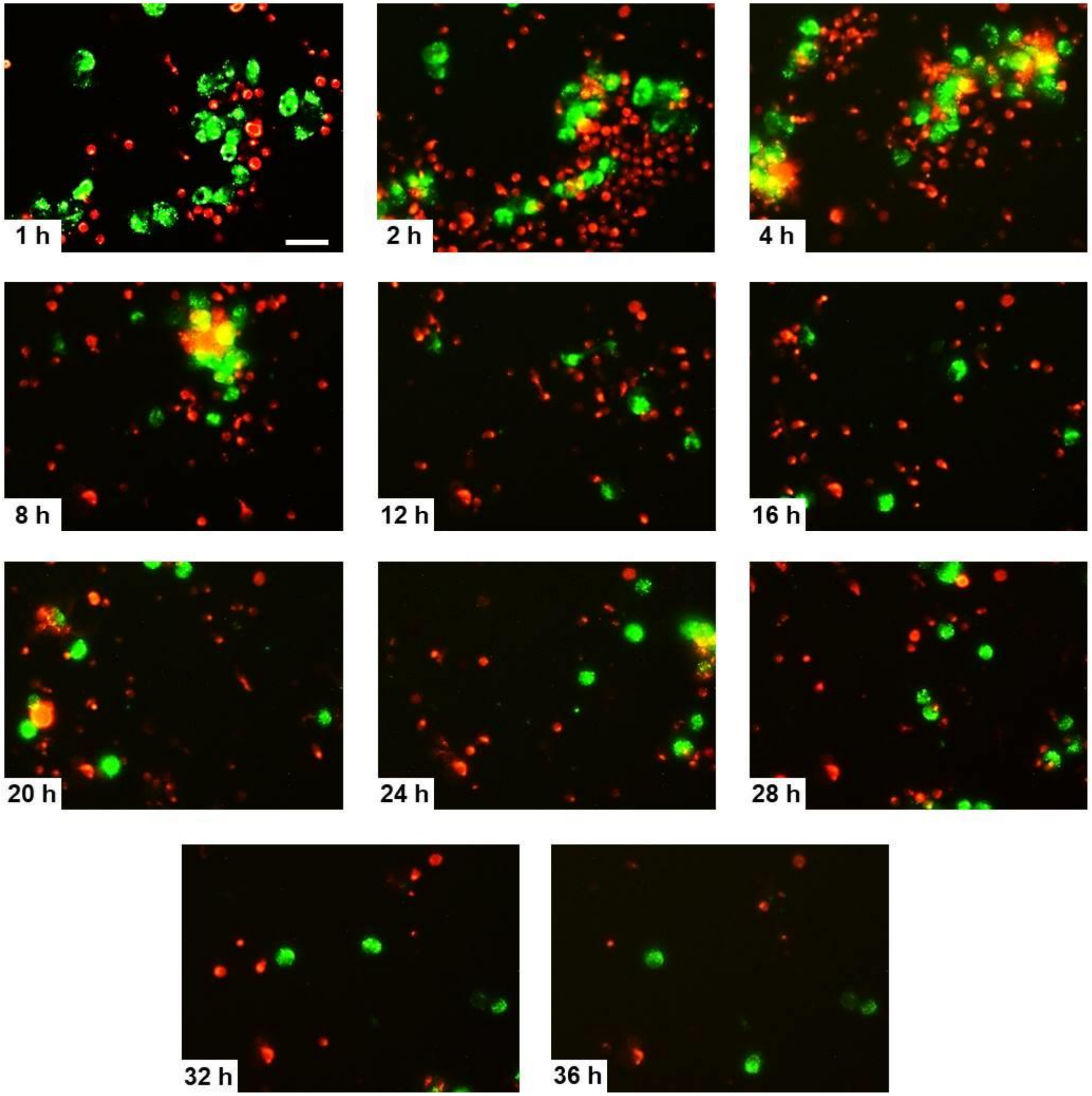

Figure S6. Time-lapse visualization of effector cell - target cell interactions *in vitro* using live microscopy. The interaction of TKD/IL-2 stimulated (S) NK cells within a PBMC preparation (red) with Hsp70^{high} LS174T target cells (green) (E:T 2.5:1) was monitored by a time-lapse imaging system at various intervals over 36 hours. The experiment was started after adding the cells to the culture. The scale bar is 50 µm.

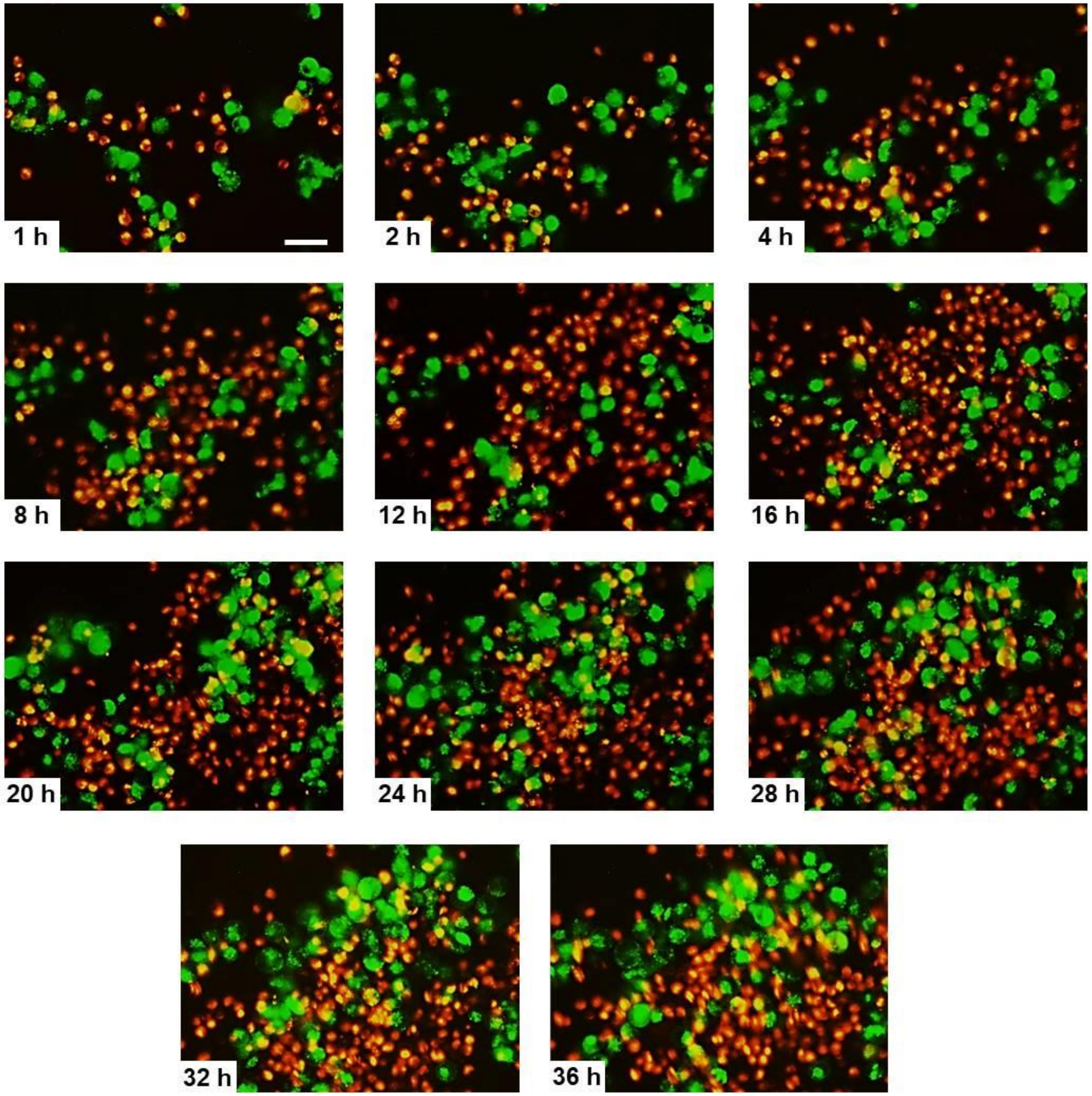

Figure S7. Time-lapse visualization of effector cell - target cell interactions *in vitro* using live microscopy. The interaction of untransduced (Unt) T cells isolated from PBMCs (red) with Hsp70^{high} LS174T target cells (green) (E:T 2.5:1) was monitored by a time-lapse imaging system at various intervals over 36 hours. The experiment was started after adding the cells to the culture. The scale bar is 50 µm.

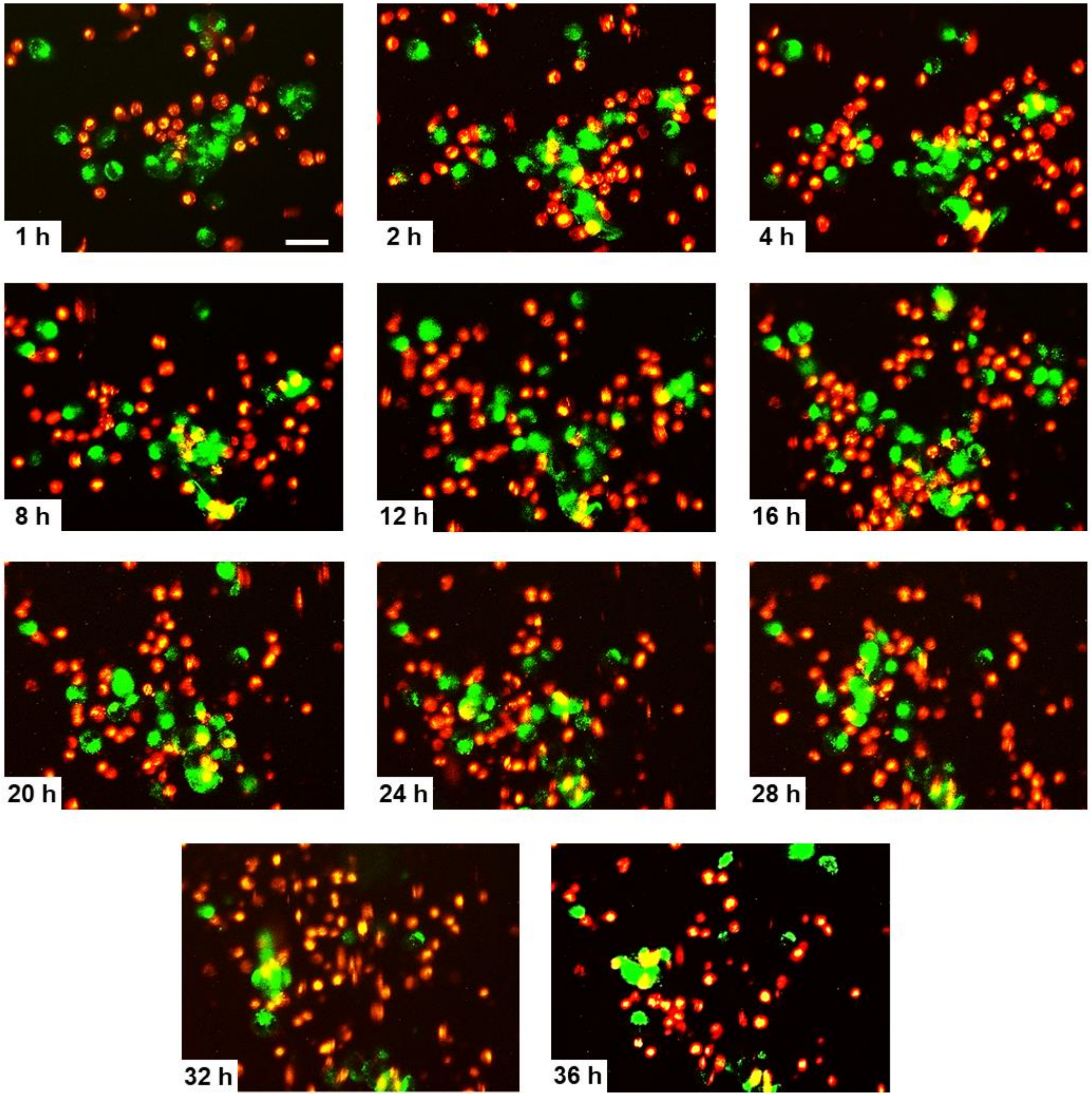

Figure S8. Time-lapse visualization of effector cell - target cell interactions *in vitro* using live microscopy. The interaction of anti-Hsp70 CAR T cells (red) with Hsp70^{high} LS174T target cells (green) (E:T 2.5:1) was monitored by a time-lapse imaging system at various intervals over 36 hours. The experiment was started after adding the cells to the culture. The scale bar is 50 µm.

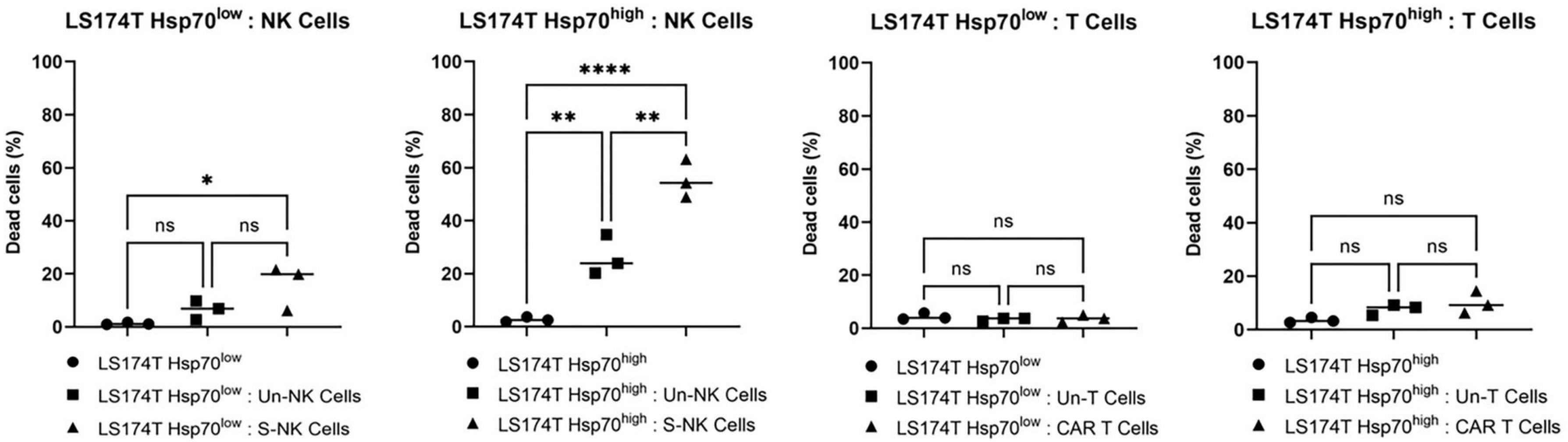

Figure S9. Flow cytometric analysis of apoptotic and necrotic cells stained by Annexin V-FITC/PI. The LS174T target cells with $Hsp70^{high}$ and $Hsp70^{low}$ expression were co-cultured with effector cells including Un-NK, S-NK, Unt-T, and CAR-T cells at an E:T ratio of 1:1 for 4 hours followed by Annexin V/PI staining and flow cytometry analysis (mean ± SD, n=3 independent donors; ns: not significant, *p≤0.05, **p≤0.01, ***p≤0.001, ****p≤0.0001).
